# How Does the Time from Intraoperative Culture Harvest to Incubation Impact Culture Positivity in Orthopaedic Surgeries? A Case–Control Study

**DOI:** 10.3390/antibiotics15090913

**Published:** 2026-09-16

**Authors:** Amir Human Hoveidaei, James K. Conway, Kyree Day, Elizabeth K. Tissingh, Janet D. Conway

**Affiliations:** 1 International Center for Limb Lengthening, Rubin Institute for Advanced Orthopedics, Sinai Hospital of Baltimore, Schoeneman Building, 2nd Floor, 2401 West Belvedere Avenue, Baltimore, MD 21215, USA; 2 Limb Reconstruction and Bone Infection Service, The Royal Ntaional Orthopaedic Hospital, Brockley Hill, London HA7 4LP, UK; 3 King’s Global Health Partnerships, School of Life Course and Population Sciences, King’s College London, London SE5 9RJ, UK

**Keywords:** bone infection, joint infection, fracture related infection, prosthetic joint infection, culture

## Abstract

Background: Orthopaedic infections require accurate microbiological diagnosis for effective treatment. This study aimed to evaluate the impact of an accelerated protocol for culture harvesting and incubation on culture positivity rates in orthopaedic surgeries, comparing it to the traditional protocol. Methods: A single-center, 1:1 matched case–control study was conducted at the Rubin Institute for Advanced Orthopaedics. Patients undergoing orthopaedic surgery with suspected infections were divided into two groups (108 patients): accelerated protocol (June–November 2024) and traditional protocol (January–June 2024). The time from operating room (OR) to incubation was significantly shorter in the accelerated group (89 min vs. 245 min, *p* < 0.001). Culture positivity rates and microorganism profiles were compared. The temporal dynamics of culture positivity were evaluated using Kaplan–Meier analysis across the entire cohort. A logistic regression analysis was carried out between the time from OR to incubation and culture positivity. Results: The accelerated protocol group had significantly higher culture positivity rates (70.4%) compared to the traditional group (48.1%) (*p* = 0.027). Microbial profiles were similar between groups. The probability of culture positivity across the entire cohort decreased by 6% for every 10-min delay after 100 min from OR. Regression analysis showed a significant inverse association between time and culture positivity (β = −0.005, *p* = 0.016). Conclusions: The accelerated protocol significantly improved culture positivity rates without compromising diagnostic performance. Reducing the time from sample collection to incubation enhances pathogen detection across all pathogen types, potentially improving outcomes in orthopaedic infection management.

## 1. Introduction

Orthopaedic infections, including periprosthetic joint infection (PJI) and fracture-related infection (FRI), pose substantial diagnostic and therapeutic challenges for surgeons and patients alike [1,2]. Accurate microbiological identification is essential for guiding targeted antimicrobial therapy and surgical management [3]. The Infectious Diseases Society of America and the American Society for Microbiology recommend prompt transport and processing of synovial fluid, tissue biopsies, and explanted prostheses, with particular attention to specimen handling and culture techniques to maximize diagnostic yield [4,5]. The FRI Consensus Guidelines stress the importance of good intra-operative sampling for the diagnosis and management of FRI [6]. Recent literature further underscores the critical role of rapid sample-to-culture workflows in preserving pathogen viability and improving detection rates, especially for biofilm-associated and fastidious organisms [7,8].

While much of the existing literature has focused on optimizing culture incubation periods and the use of enriched media or sonication fluid culture for PJI and FRI, there remains a gap regarding the impact of accelerating the time interval from intraoperative sample harvest to culture initiation [4,8,9]. Delays in specimen processing have been shown to reduce culture positivity, particularly for organisms with limited environmental stability [7,8]. However, to the best of our knowledge, no studies have systematically evaluated the clinical effect of protocol-driven acceleration of sample transport and laboratory handoff in real-world surgical cohorts in orthopeadics.

The present study aims to address this gap by comparing a recently implemented accelerated protocol at our center, designed to minimize the time from intraoperative sample harvest to culture initiation, with the traditional protocol. The study cohort includes patients undergoing orthopaedic surgery for suspected PJI or FRI/osteomyelitis. The primary objectives are to compare (1) culture positivity rates and diagnostic performance, (2) microorganism profiles, and (3) the time-dependent probability of culture positivity for the entire cohort.

## 2. Methods

### 2.1. Overview

This single-center case–control study was conducted at the Rubin Institute for Advanced Orthopaedics, Maryland, with approval from the Institutional Review Board (IRB; code: 2024-041; Date: 1 August 2024). The study aimed to assess the impact of a newly implemented accelerated protocol for culture harvesting and incubation, introduced in mid-June 2024. All samples were tissue specimens harvested at the earliest possible point at the beginning of the procedure and immediately transported to the microbiology lab, where processing was initiated as quickly as possible. Lab technicians recorded key times, including sample receipt, order entry in the hospital system (Cerner), plating initiation, and incubation. Based on the available data for the historical group, we have presented the aligned data in the manuscript to ensure a more accurate and practical comparison: the OR side, referring to the time from case start in the OR to lab arrival (OR-to-lab), and the lab side, referring to the time from sample receipt at the lab to incubation start (lab-to-incubation). The sum of these two components makes up the total OR-to-incubation time.

### 2.2. Study Design and Population

Data were collected from the microbiology laboratory of the hospital. All surgical cases involving PJI or FRI/osteomyelitis (confirmed by MSIS [10] and FRI Consensus [6] criteria, respectively) performed by the division head were included if intraoperative tissue samples were sent for microbiological analysis. Patients were divided into two groups, including the accelerated protocol group, which included cases from mid-June to November 2024. To ensure comparability between the groups, all cases from the accelerated protocol group were included and retrospectively matched with historical cases based on the type of surgery (PJI vs. FRI/osteomyelitis). Matching was performed by selecting the first available case from the historical group, going back from mid-June to January 2024, that met the same surgical criteria (PJI vs. FRI/osteomyelitis) to achieve a 1:1 match. The groups were comparable regarding the distribution of upper and lower FRI cases (*p* = 1.000). The control group consisted of cases from this historical period, which followed the traditional protocol.

In the historical control group, no specific emphasis was placed on expediting sample transport to the laboratory or initiating incubation. In the accelerated group, all personnel were instructed to complete their respective tasks as promptly as feasible, without predefined time limits, to maintain the practicality and real-world applicability of the workflow.

### 2.3. Sample Collection

All patients had received antibiotic therapy prior to surgery. Samples obtained from outpatient procedures, superficial and synovial fluid aspirations including both preoperative and intraoperative, and bone samples were excluded from the analysis. According to the division protocol, the surgeon collects intraoperative cultures during the first few minutes of the procedure as the initial stage of the operation. Tissue samples were collected intraoperatively at the discretion of the surgeon based on gross appearance, following the division’s standard protocol of obtaining five cultures per surgery, informed by international guidelines [11,12,13]. All specimens were collected in sterile specimen cups, and no swabs were utilized. All samples were routinely sent for aerobic and anaerobic cultures. Cultures were also sent for fungal and acid-fast bacilli (AFB) testing and no sonication was done. Standard incubation was five days, with cultures held for up to 2 weeks if no growth was detected at the initial 5-day period to capture slow-growing organisms.

Specimen collection followed a standardized protocol consistent with current guidelines. Tissue samples were obtained at the beginning of surgery, before irrigation or debridement, using clean instruments and placed directly into sterile containers without environmental contact to minimize contamination. No swab cultures were used, as tissue specimens are the recommended standard for orthopaedic infection diagnosis. A minimum of five individually labeled tissue samples were collected per case and transported at room temperature for aerobic and anaerobic culture.

### 2.4. Outcome Measures

The study’s outcome measures included the culture positivity rate, defined as the identification of a confirmed pathogen, requiring growth of the same organism from at least two independent tissue specimens. Contaminated samples, such as those yielding only skin flora, were excluded. Each patient’s final culture result was categorized as positive or negative. The microorganism profile of culture-positive samples was examined, comparing bacterial and fungal species between traditional and accelerated protocols. Finally, logistic regression analysis was used to assess the relationship between the time from OR to incubation and the likelihood of a positive culture result across the entire cohort.

### 2.5. Statistical Analysis

All statistical analyses were performed using R (Version 4.5.0, R Foundation for Statistical Computing, Vienna, Austria). McNemar’s test compared culture positivity rates and sex distribution between groups. Paired *t*-tests compared age distributions. The distribution of microorganisms was compared using Chi-squared tests for Gram-positive cocci, Gram-positive rods, Gram-negative rods, and polymicrobial infections, and Fisher’s exact test for anaerobes and fungi. The Wilcoxon signed-rank test compared time from OR to incubation. Kaplan–Meier survival curves were created using the “survival” and “survminer” libraries. Kaplan–Meier survival analysis was used to model time from OR to incubation, enabling assessment of the temporal pattern of culture growth and quantification of changes in positivity probability. The Kaplan–Meier method was used to model the time-dependent probability of culture positivity across the entire cohort. In the present study, the time variable was the interval from OR to incubation (in minutes), and the event was defined as a positive culture result. Patients whose cultures remained negative at the end of the standard incubation period were treated as right-censored observations. Logistic regression analyses were conducted for both the traditional and accelerated protocols to assess the relationship between the time from OR to incubation (as the independent variable) and culture positivity. Statistical significance was set at *p* < 0.05.

## 3. Result

### 3.1. Patient Demographics

**Figure 1:**
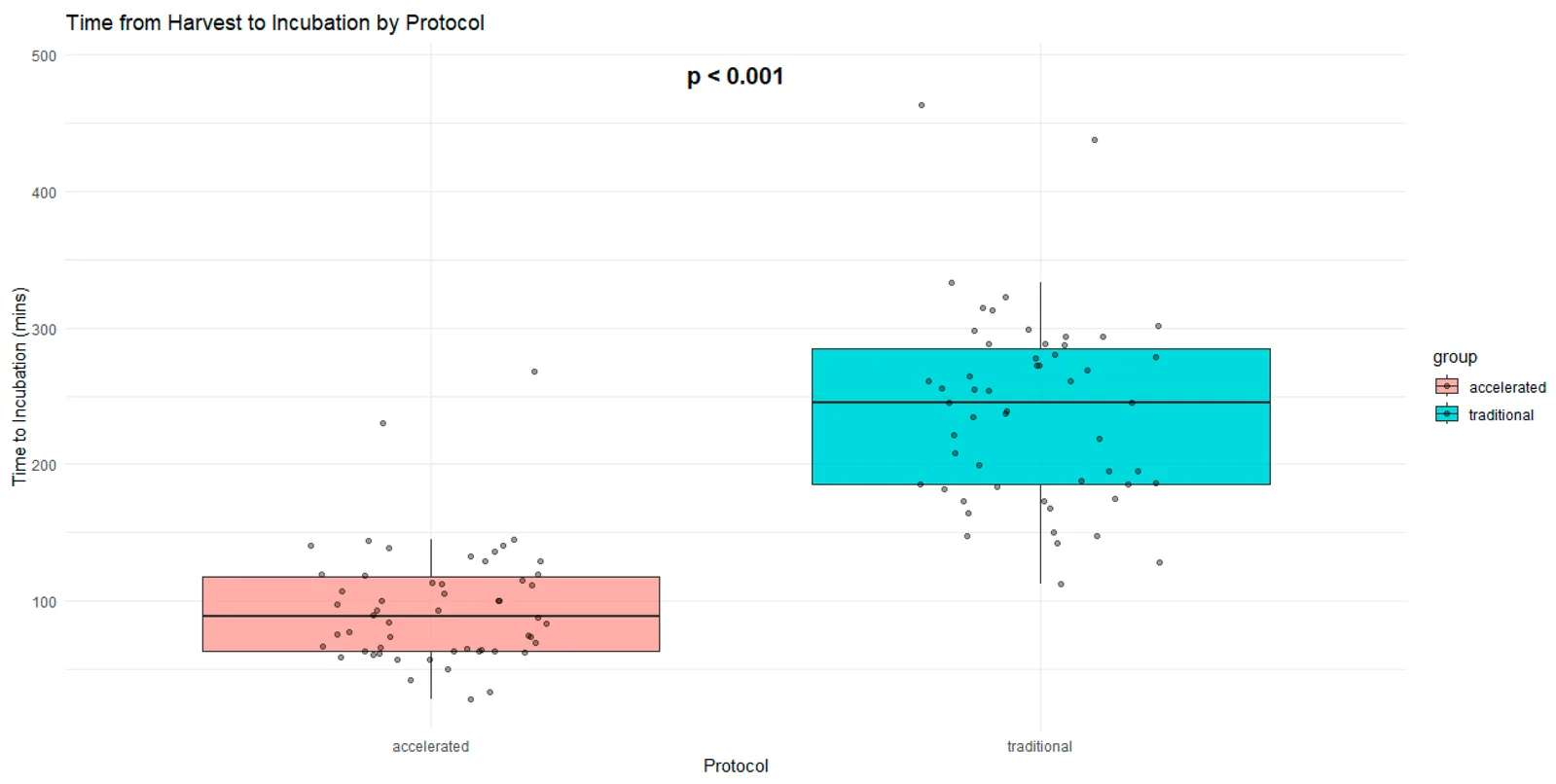
Time difference between the groups (operating room to incubation).

Statistical analysis revealed that the time from the OR to incubation was significantly shorter in the accelerated group compared to the traditional group, with a median of 89.00 min (IQR: 63.25–118.00) in the accelerated group versus a median of 245.00 min (IQR: 185.0–285.2) in the traditional group (*p* < 0.001) (Figure 1).

Specifically, the mean time from the OR to incubation was 95.4 (±43.3) minutes for the accelerated group and 240.0 (±70.3) minutes for the traditional group. The mean time from the OR to the lab was 47.5 (±36.9) minutes for the accelerated group compared to 116.9 (±62.2) minutes for the traditional group (*p* < 0.001). The lab-to-incubation period was 48.1 (±25.7) minutes in the accelerated group and 123.3 (±41.6) minutes in the traditional group (*p* < 0.001).

Initially, 55 patients were enrolled in the accelerated group; however, one patient was excluded due to an incorrect label in Cerner, where it was listed as an intra-operative tissue culture but was actually synovial fluid, resulting in 54 patients in the case group. After 1:1 matching, 54 patients from the traditional control group were included, bringing the total number of patients in the study to 108. Both groups consisted of 32 joint infections and 22 FRI/osteomyelitis cases. The sex distribution was similar between groups (*p* = 0.290): accelerated group, 27 (50%) males; traditional group, 33 (61.1%) males. The mean age was 62.93 years (±13.8) in the accelerated group and 60.87 years (±15.2) in the traditional group, with no significant difference (*p* = 0.464).

### 3.2. Culture Positivity and Regression Analysis

In the accelerated group, 38 out of 54 patients (70.4%) had positive cultures, while 26 out of 54 patients (48.1%) in the traditional group had positive cultures. The difference in culture positivity rates between the two groups was statistically significant, with a *p*-value of 0.027.

### 3.3. Microorganisms Distribution

**Figure 2:**
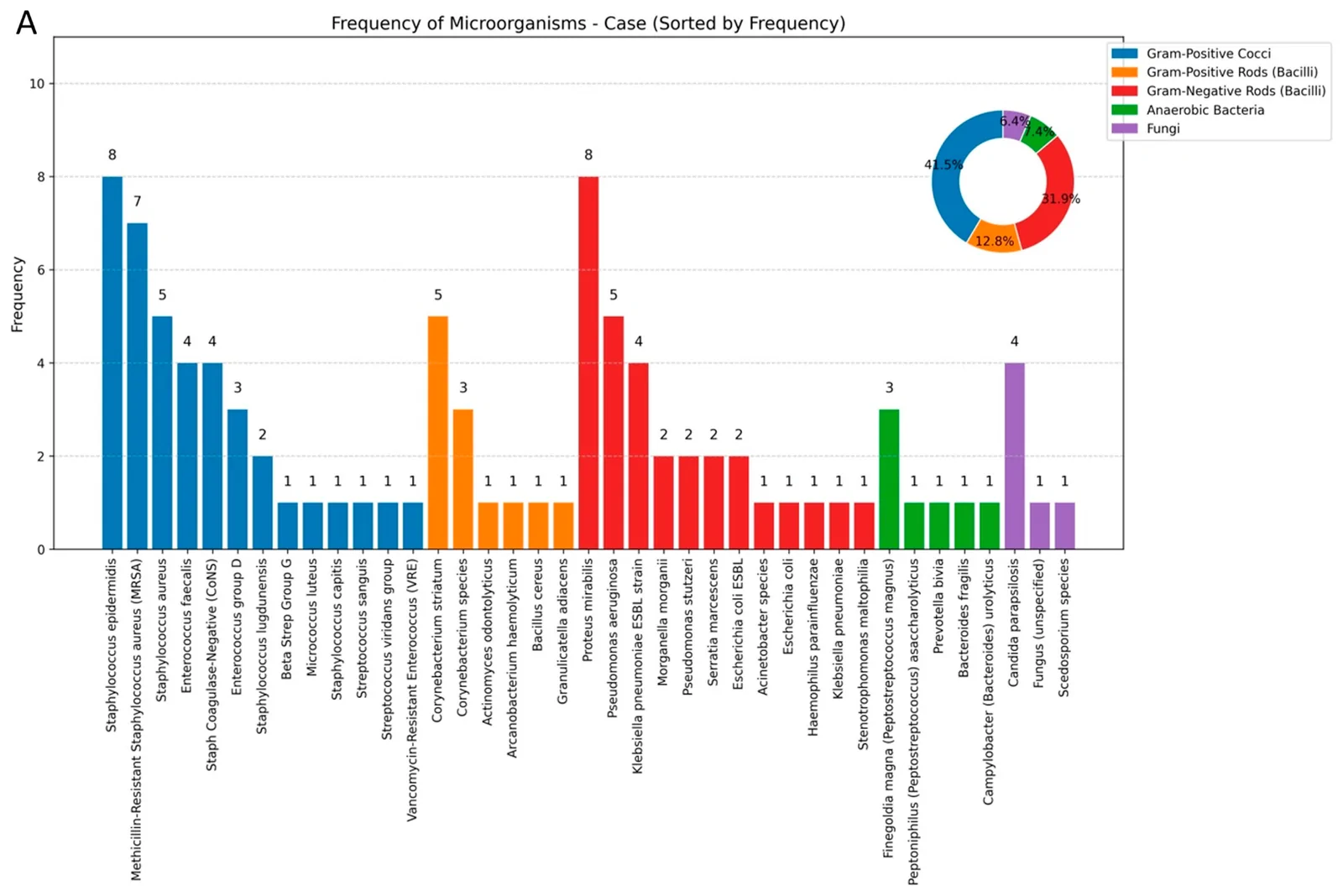

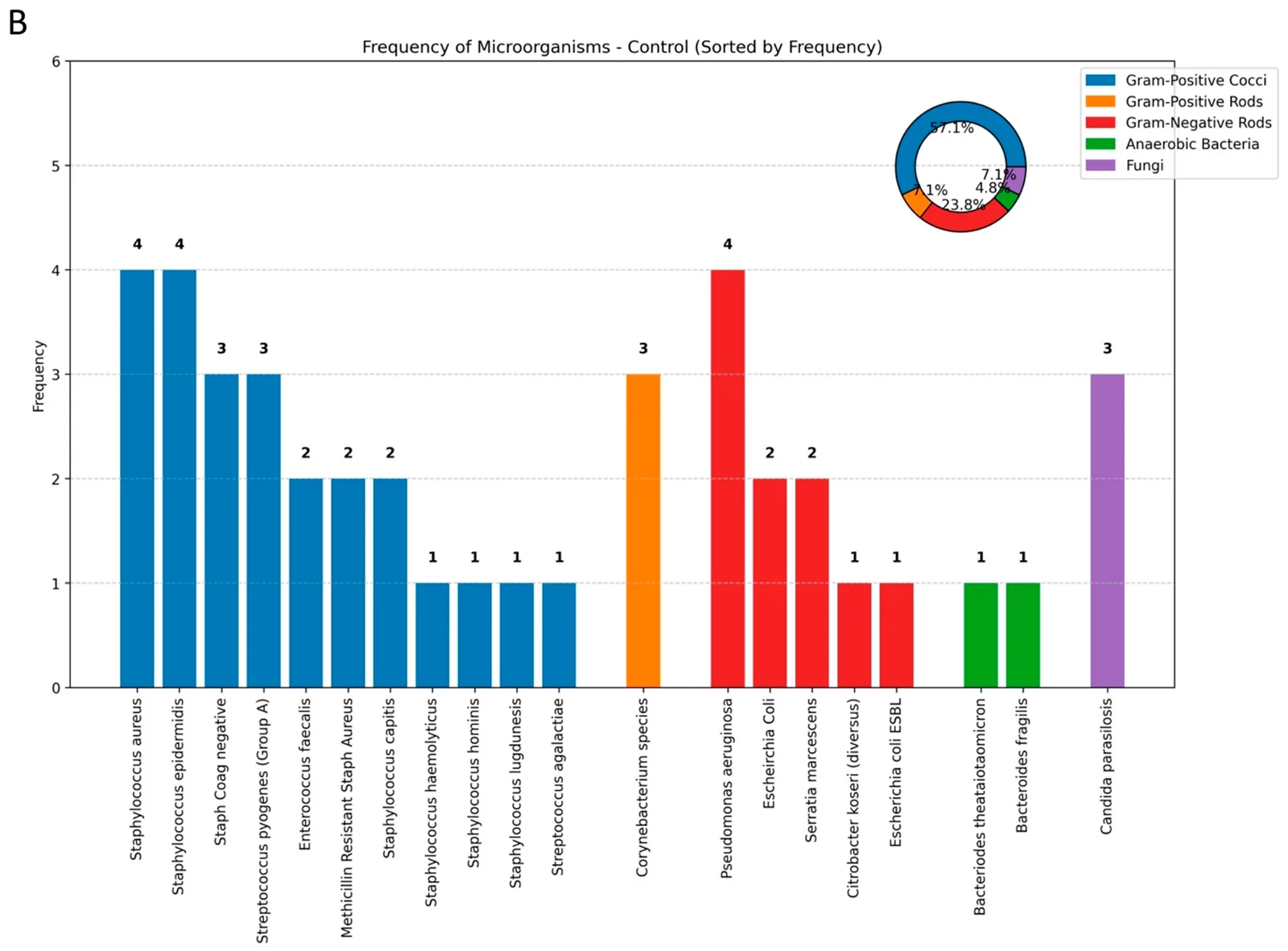
Microorganism profile in (**A**) cases (accelerated cohort); (**B**) controls (traditional cohort).

The microorganism profiles for both groups were plotted to highlight the differences in the types of microorganisms detected (Figure 2A,B).

A total of 39 types of microorganisms were detected in the accelerated group, compared to only 20 types in the traditional group, with no contamination observed. The details of the species are shown in Figure 2A,B.

When comparing the microorganism groups (Gram-positive cocci, Gram-positive rods, Gram-negative rods, anaerobes, and fungi), no significant differences were observed between the accelerated and traditional cohorts (*p* = 0.361, *p* = 0.766, *p* = 1, *p* = 0.489, *p* = 1, respectively). Polymicrobial infections were seen in 21 (55.2%) of 38 cases in the accelerated group and 11 (42.3%) of 26 cases in the traditional group, with no significant difference between the two groups (*p* = 0.445).

### 3.4. Time-Dependent Probability of Culture Positivity

**Figure 3:**
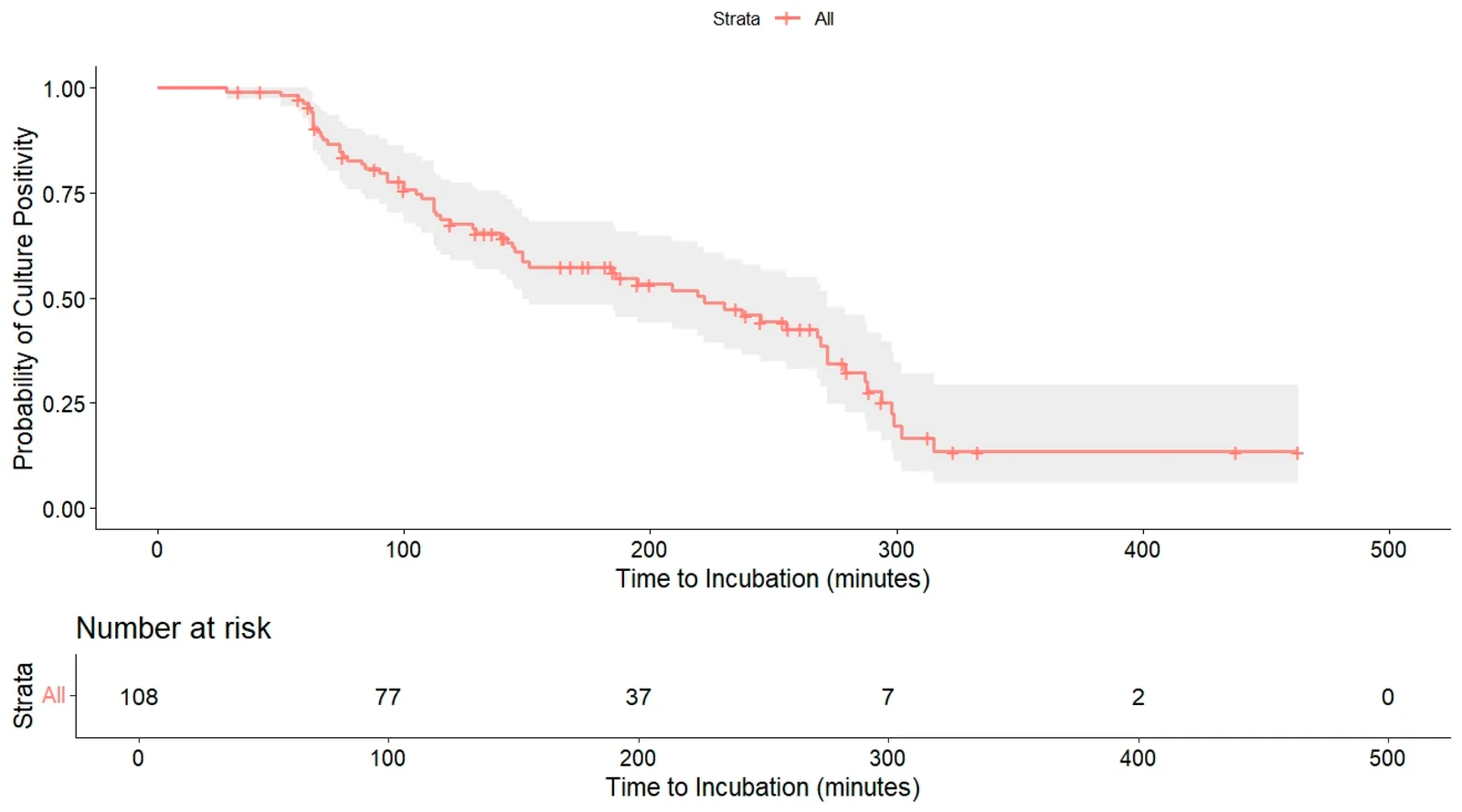
Kaplan–Meier analysis for culture positivity in the entire cohort (based on the time from operating room to incubation).

Pooling the data from all 108 case and control groups revealed that the median time to culture positivity was 222 min for the entire population, representing the point at which 50% of cultures yielded growth. According to the Kaplan–Meier curve, after 100 min, the probability of culture positivity decreases by an average of 6% for every subsequent 10-min delay (Figure 3).

Regression analysis demonstrated that the association between time and culture positivity was statistically significant (β = −0.005, *p* = 0.016).

## 4. Discussion

The accelerated protocol was implemented without additional resources. The surgical and nursing teams prioritized immediate collection and handoff of specimens, with transport staff notified to retrieve the samples as soon as they were harvested. All paperwork was completed at the time of specimen collection, enabling rapid delivery to the microbiology laboratory. This approach demonstrates that an accelerated workflow is feasible in routine practice without additional staffing or equipment.

The primary finding of this study is that implementation of an accelerated protocol, reducing the time from operation to incubation from a median of 245 min to 89 min, was associated with a higher rate of culture positivity in orthopaedic infections. This result aligns with the pathophysiological understanding that prompt specimen processing preserves microbial viability and reduces the risk of false-negative cultures due to bacterial death or overgrowth of contaminants [4,14,15]. The robust design of the present study, with strict matching and lack of contaminated samples, strengthens the evidence that accelerated protocols are beneficial. Clinically, these findings underscore the importance of optimizing laboratory workflows to ensure timely incubation, which may improve pathogen detection and guide targeted antimicrobial therapy, ultimately enhancing patient outcomes in orthopaedic infection management [4,5,16,17].

Analysis of microorganism profiles showed that the accelerated protocol did not alter the spectrum of detected pathogens, with similar distributions of Gram-positive cocci, Gram-negative rods, anaerobes, and fungi across both groups. While previous studies have highlighted the recovery of most clinically relevant pathogens within the first five days of incubation, none have specifically examined the impact of transport and processing time on culture yield [9,18,19,20,21]. This emphasizes the importance of rapid specimen handling to improve diagnostic efficiency while maintaining standard incubation periods, which has notable implications for laboratory practice.

This study found that after 100 min, the probability of culture positivity decreases by 6% for every 10-min delay. This aligns with previous studies showing reduced culture yields with delays [14,22]. Deas et al. [14] observed a 0.997-fold decrease for every hour between blood collection and incubation. Similarly, Venturelli et al. [22] demonstrated a 0.3% reduction in positivity rate for each hour of delay in blood culture loading in adult septic patients. Differences in sample type and patient settings may explain variations in our results. The increase in culture positivity with faster harvest-to-incubation times has notable clinical implications for orthopaedic infections. Notably, the accelerated protocol did not show a higher rate of fastidious or anaerobic organisms, suggesting it benefits a broad range of pathogens. These findings support closer collaboration between surgical and laboratory teams to optimize specimen handling, early pathogen identification, and targeted therapy, reducing unnecessary broad-spectrum antibiotic use [16,23,24]. Accelerating protocols may also improve laboratory efficiency and patient outcomes in orthopaedic infection care [5,23].

A key strength of this study is its robust case–control design with strict 1:1 matching, ensuring a balanced comparison of accelerated and traditional protocols. The clear outcome measures, including culture positivity rates and microorganism profiling, provided a comprehensive evaluation. However, a limitation is the single-center design, which may limit generalizability. The use of a historical control group may have introduced selection and temporal biases. Additionally, while key confounding factors were controlled for, unmeasured variables, such as patient-specific factors or variations in tissue sample handling, could still influence the results. Findings within subgroups, such as the association between time and the type of microorganisms, can be more accurately reported in future multicenter studies with different lab protocols and larger sample sizes, especially since this is the first study of its kind and calculating the sample size based on prior literature is challenging. Additionally, the duration and spectrum of preoperative antibiotic therapy was not consistently available in the medical records and therefore could not be analyzed. Differences in case complexity and infection chronicity between groups may have introduced potential confounding. Lastly, a limitation of our study is that, for our control historical group, we only had case start time and not harvest time. As a result, we used case start time for both groups in the OR-to-incubation time calculation. The ideal approach would have been to measure the time from harvest to incubation for both groups, which would have required prospective data collection for the control group. However, we could not implement intentional delays for one patient group, as this would not have been ethically permissible.

In conclusion, the accelerated culture harvesting and incubation protocol reduced incubation time and increased culture positivity across all pathogens. After 100 min, culture positivity decreased by 6% for every 10-min delay. Optimizing the time from sample collection to incubation can improve pathogen detection and ultimately enhance patient outcomes in orthopaedic infection management.

## Data Availability

The data presented in this study are available from the corresponding author upon reasonable request due to privacy and ethical considerations.
